# Supplementary material for: Identification of photoperiod-induced specific miRNAs in the adrenal glands of Sunite sheep (Ovis aries)
Source: Front Vet Sci. 2022 Jul 22;9:888207. doi: 10.3389/fvets.2022.888207 (PMC9354845; doi:10.3389/fvets.2022.888207)
Supplement: Supplementary file 1 [file Data_Sheet_1.ZIP › Supplementary file/miRDeep2 file.zip!/miRDeep2 file/Know_Structure/LP42A2/oar-mir-543.pdf]

miRBase precursor : oar-mir-543
Total read count : 3468
oar-miR-543-5p read count : 8
oar-miR-543-3p read count : 3438
remaining reads : 22

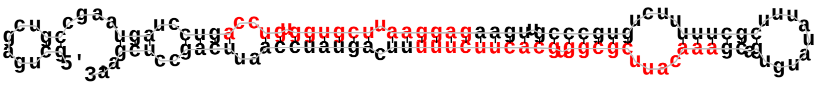

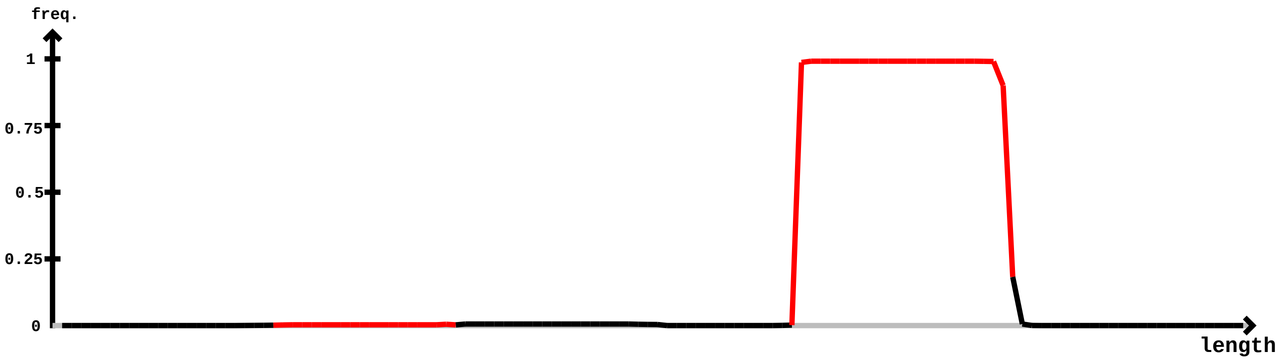


```
                                                                                         oar-miR-543-3p
                      oar-miR-543-5p
5'- gcugagcugccgaaugauccugaccuguggugcuuaaggagaaguugcccgugucuuuuucgcuuuauaugugacgaaacauucgcggugcacuucuuuuucaguauccaauucagccucgaa  -3'   exp
    ((......)).....((..((((..((.((((((.((((((((((.(((((((....((((((.........).)))))....))))).)))))))))))).))))))))..))))..))...  reads   mm
    ..................ccugaccuguggugcuuaagga...................................................................................  1   0
    ...................cugaccuguggugcuuaagga...................................................................................  1   0
    ....................ugaccuguggugcuuaagga...................................................................................  1   0
    .....................gaccuguggugcuuaagga...................................................................................  2   0
    ......................accuguggugcuuaagga...................................................................................  1   0
    ......................accuguggugcuuaaggag..................................................................................  2   0
    .......................ccuguggugcuuaagga...................................................................................  2   0
    .......................................agaaguugcccgugucuuuu................................................................  3   0
    .......................................agaaguugcccgugucuuuuu...............................................................  2   0
    .......................................agaaguugcccgugucuuuuucg.............................................................  2   0
    .........................................aaguugcccgugucuuuuuc..............................................................  1   0
    .........................................aaguugcccgugucuuuuucg.............................................................  12  0
    ..........................................................................cgaaacauucgcggugcacuuc...........................  1   0
    ..........................................................................cgaaacauucgcggugcacuucu..........................  1   0
    ...........................................................................gaaacauucgcggugcacuucu..........................  4   0
    ............................................................................aaacauucgcggugcacuu............................  2   0
    ............................................................................aaacauucgcggugcacuuc...........................  1   0
    ............................................................................aaacauucgcggugcacuucu..........................  307  0
    ............................................................................aaacauucgcggugcacuucuu.........................  2481 0
    ............................................................................aaacauucgcggugcacuucuuu........................  605  0
    ............................................................................aaacauucgcggugcacuucuuuu.......................  19   0
    ............................................................................aaacauucgcggugcacuucuuuuu......................  1    0
    .............................................................................aacauucgcggugcacuucuu.........................  9    0
    .............................................................................aacauucgcggugcacuucuuu........................  7    0
```
